# Implicit Bias and Patient Care: Mitigating Bias, Preventing Harm

**DOI:** 10.15766/mep_2374-8265.11343

**Published:** 2023-09-19

**Authors:** Hannah Barber Doucet, Taneisha Wilson, Lauren Vrablik, Robyn Wing

**Affiliations:** 1 Assistant Professor, Division of Pediatric Emergency Medicine, Department of Pediatrics, Boston University School of Medicine; 2 Assistant Professor, Department of Emergency Medicine, Warren Alpert Medical School of Brown University; 3 Third-Year Fellow, Division of Pediatric Emergency Medicine, Department of Emergency Medicine, Warren Alpert Medical School of Brown University; 4 Associate Professor, Division of Pediatric Emergency Medicine, Departments of Emergency Medicine and Pediatrics, Warren Alpert Medical School of Brown University and Rhode Island Hospital/Hasbro Children's Hospital, and Director of Pediatric Simulation, Lifespan Medical Simulation Center

**Keywords:** Implicit Bias, Nonaccidental Trauma, Emergency Medicine, Health Equity, Pediatric Emergency Medicine, Simulation, Anti-racism, Diversity, Equity, Inclusion

## Abstract

**Introduction:**

Simulation is a valuable and novel tool in the expanding approach to racism and bias education for medical practitioners. We present a simulation case focused on identifying and addressing the implicit bias of a consultant to teach bias mitigation skills and limit harm to patients and families.

**Methods:**

Learners were presented with a case of a classic toddler's fracture in an African American child. The learners interacted with an orthopedic resident who insisted on child welfare involvement, with nonspecific and increasingly biased concerns about the child/family. The learners were expected to identify that this case was not concerning for nonaccidental trauma and that the orthopedic resident was demonstrating bias. They were expected to communicate with both the resident and the parent effectively to defuse the situation and prevent harm from reaching the family. A debrief and an anonymous survey followed the case.

**Results:**

Seventy-five learners participated, including pediatric and emergency medicine residents, fellows, attendings, and medical students. After the case, the majority of learners expressed confidence that they could recognize racial bias in the care of a patient (90%), ensure patient care was not influenced by racial bias (88%), and utilize a tool to frame a concern about bias (79%).

**Discussion:**

Participants felt that this simulation was relevant and effective and overall left the experience feeling confident in their abilities to identify and manage racially biased patient care. This anti-racist simulation offers an important skill-building opportunity that has been well received by learners.

## Educational Objectives

By the end of this session, learners will be able to:

1. Have confidence in identifying situations where implicit bias and racism may impact medical decision-making.
2. Express concern about racial bias using the ACT (affirm-counter-transform) tool.
3. Express confidence in their ability to prevent biased assessments from impacting patient care decisions.
4. Identify appropriate and inappropriate medical scenarios in which to have concern for nonaccidental trauma.

## Introduction

Amidst years of evidence that the care provided to patients is inequitable,^1,2^ the need to address racism in medicine head-on has created a new standard for all domains of medicine, including medical education. The individual decisions of providers are often influenced by bias, leading to substandard care and uncomfortable patient interactions.^3–6^ In pediatric emergency medicine (PEM), racial disparities have been described in several aspects of care, including, but not limited to, treatment of pain for children with appendicitis, utilization of imaging, and antibiotic prescriptions.^7–9^ Both in the emergency department and outside it, there is a racial disparity in nonaccidental trauma allegations, investigations, and outcomes.^4,10–12^ Black children are more frequently referred for investigation and removed from their family's care across the country. There remains debate regarding the multifactorial nature of this racial disproportionality, but one of these factors is likely the racial bias underlying individual decision-making. At least one study has demonstrated that standardizing medical decision-making regarding abuse workups in pediatric hospitals erases the racial disparity in abuse allegations by medical providers.^13^ This strongly suggests that medical providers, left to their own gestalt of when to be concerned about nonaccidental trauma, can be influenced by racial implicit bias.

When addressing individual racism and implicit bias, there are several potential domains to consider, including the bias of the learners themselves, the bias of team members, and the bias of patients. Each of these domains contains unique challenges and the need for overlapping but distinct skill sets. It is imperative for all medical providers to be aware of how racial bias impacts care and be prepared to address it as part of a larger effort to undo racial health disparities and improve the patient and family medical experience. Emergency department physicians in particular must be adept at utilizing such skills in a fast-paced environment that often includes a rotating group of team members and consultants.

A variety of educational resources have been developed to combat provider bias and racism.^14–17^ Skill building for uncomfortable scenarios can be challenging in a lecture or other more theoretical format. Simulation offers a new potential modality for combating racial bias. Addressing the bias of others on the medical team can be difficult in the moment, and early learners often describe a deer-in-the-headlights panic, or a passivity, when faced with these scenarios. This discomfort makes such an interaction ripe for simulation, where learners can practice their skills in a realistically stressful but safe learning environment. A few authors have described the use of standardized patients to improve learning about racial bias,^18–20^ and this modality has successfully been used in the past to teach cultural competency.^21,22^ However, there are few published studies on the use of simulation to address racism and implicit bias, as well as no similar simulation publications in *MedEdPORTAL*.

We present a simulation case focused on identifying and addressing the implicit bias of a consultant to teach bias mitigation skills and limit harm to patients and families. This simulation case is part of a series from our larger educational initiative, Discussing Anti-racism and Equity in Emergency Medicine (DARE EM). The DARE EM curriculum has been developed to improve knowledge and skills related to anti-racism in medicine. This curriculum includes simulation sessions, lectures, and reading groups dedicated to anti-racism and equity topics. This simulation can be used independently or in conjunction with other sessions from the DARE EM curriculum or other similarly focused curricula.

## Methods

### Development

This case was developed by PEM and emergency medicine (EM) physicians to teach bias mitigation skills. It was initially developed for PEM, EM, and pediatric learners, including residents, fellows, medical students, and faculty. Some EM learners received the simulation as part of the DARE EM curriculum; the remainder participated in the simulation as a stand-alone educational session. Prerequisite knowledge included identification of a toddler's fracture and knowledge regarding pediatric findings that would (and would not) be concerning for nonaccidental trauma.

### Equipment/Environment

This case (Appendix A) was run at a medical simulation center, which emulated a standard clinical space, as well as in a classroom. The learners were introduced to the case by a signing-out provider, played by a facilitator, who utilized a computer to show the patient's medical chart front sheet, a typed medical note, and the patient's X-ray while introducing the case (Appendices B and C). The case otherwise required a toddler-sized mannequin with dark skin on a stretcher, a curtain or room divider to split the simulation space into two rooms, and the personnel described below.

### Personnel

The simulation required a minimum of three to four personnel: the orthopedic resident (biased consultant), the patient's parent (specifically Black/African American), the nurse, and the signing-out provider. With only three personnel, the same person could stand in as the signing-out provider and switch to the nurse role, as these had no overlap. The primary facilitator could play the nurse role, as they were available to answer questions and gently redirect the learner if needed. The role of the parent and potentially that of the orthopedic resident could be done by a standardized patient. Due to COVID-19 restrictions in place at our institution that precluded the use of standardized patients, these roles were played by EM and PEM faculty instructors. See Appendix D for the standardized participant transcripts. Each case utilized one direct participant with several observers; when the case was run with residents, a resident of second year or above was selected because they would more likely possess the prerequisite knowledge needed to have a greater focus on the bias mitigation portion of the case rather than the medical learning points.

### Implementation

The simulation was performed during scheduled simulation education time for PEM, EM, and pediatric learners between August 2021 and January 2022. The simulated case was completed in approximately 10 minutes, and the debrief ran for an additional 20 minutes. The scenario began with the learner receiving sign-out from a provider in the emergency department leaving their shift (in this case, a facilitator/standardized participant). The standardized participant introduced the patient by presenting the electronic medical record screen. This record included the patient's photo to signal his race to the learner. The sign-out also included the patient's age, sex, and chief complaint—an 18-month-old male with leg pain after going down a slide in his mother's lap. The history stated that the patient's foot was caught on the side of the slide and his leg twisted. The presented X-rays showed a spiral fracture of the left tibia consistent with a toddler's fracture (Appendix B). The provider's note was shown so that the learner could return to it if needed (Appendix C). The learner was informed that the child was awaiting a postsedation assessment after cast placement. The learner needed only to check with the orthopedic resident to ascertain follow-up and discharge the patient.

The signing-out physician then left, and the orthopedic resident entered. The resident gave the learner the appropriate discharge information and asked them to call the Department of Children, Youth and Families (DCYF, the state's child protective services department) regarding the fracture. From here, a number of branch points in communication and actions existed, with the learner ultimately having to choose whether to call DCYF or not prior to the case ending. The learner could immediately ask further questions of the orthopedic resident (as the case of a toddler's fracture sustained while going down a slide would be common and should not, in isolation, raise any concerns for nonaccidental trauma), immediately agree, or choose to evaluate the patient and mother themselves to obtain more information. When questioned or challenged, the orthopedic resident increasingly revealed their bias, although never explicitly mentioned race. If the learner immediately agreed to call DCYF at the beginning of the case, the nurse prompted them to see the patient and mother. The full case is outlined in Appendix A.

### Debriefing

To begin debriefing, ground rules were reviewed to establish a safe and respectful learning environment. Learners were asked to summarize the case and to reflect on their experience participating in and observing the simulation. Pertinent medical learning points were then reviewed, including the definition of a toddler's fracture and what findings in the emergency department would be concerning for nonaccidental trauma. The debrief next turned to a discussion of racism and implicit bias, both specific to the case of nonaccidental trauma and more broadly in pediatric and EM practice. Facilitators of this portion of the debrief included content experts in the field of diversity, equity, and inclusion (DEI). For participant groups less familiar with this material, a slide set was reviewed to outline basic concepts (Appendix E). For participant groups that had prior exposure to these topics as part of DARE EM, a group discussion was facilitated without slides. For all groups, the affirm-counter-transform (ACT) tool^23^ was introduced and demonstrated. Participants were instructed to practice using this tool with a partner or small group. Debriefing materials are presented in Appendix F.

### Assessment

A postsimulation survey (Appendix G) was created to evaluate the effectiveness of the simulated case in achieving the educational objectives as perceived by the learners and to obtain learner impressions of the simulation. This survey utilized 5-point Likert scales as well as qualitative open-ended reflections on major takeaways from the case. Qualitative responses were coded using deductive thematic analysis. All learners were able to participate in the survey upon completion of the case and debrief.

## Results

Seventy-five learners participated and completed surveys. Nine participants were actively involved in the case scenario while the others observed and then actively participated in the debrief. Forty-six were EM residents, 10 were pediatrics residents, three were PEM fellows, eight were attendings, and eight were medical students on their EM or pediatrics rotations. EM residents were drawn from two different local EM residency programs.

The majority of participants agreed or strongly agreed that the case was relevant to their work, was realistic, and was effective in teaching bias communication skills (Table 1). The majority also agreed that the debrief created a safe environment and promoted reflection and team discussion.

**Table 1.**
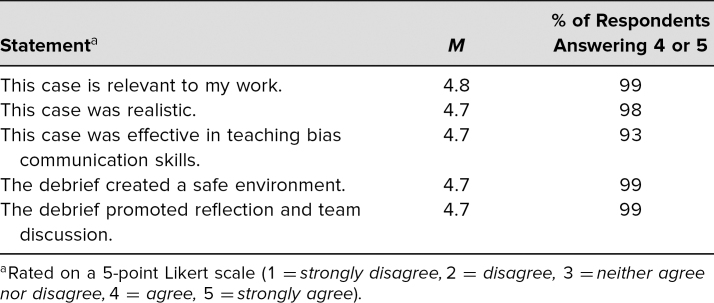
Participant Feedback on Relevance to Training and Simulation Case Quality

Following the case, 90% felt confident that they could identify situations where implicit bias and racism might impact medical decision-making. Seventy-nine percent were confident that they could express concern about racial bias using the ACT tool. Finally, 88% felt confident that they could prevent biased assessments from impacting patient care decisions (Table 2).

**Table 2.**
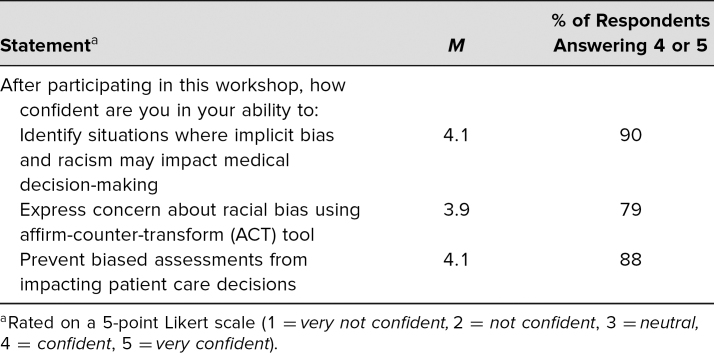
Participant Feedback on Self-Confidence in Relation to Learning Objectives

Forty-nine respondents provided qualitative responses. When participants were asked about takeaways from the case, major themes included bias assessment, bias mitigation, and racism in medicine (Table 3). A minority of participants reflected on the themes of improving communication skills with families or specific medical learning points.

**Table 3.**
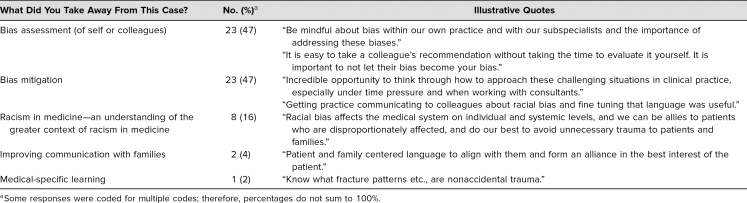
Qualitative Feedback: Takeaways From the Simulation Case (*N* = 49)

## Discussion

Simulation is a valuable tool in the expanding approach to racism and bias education for medical practitioners. This simulation was created to improve bias mitigation skills in a realistic and safe learning environment. Participants felt that the simulation was relevant and effective, and overall, they left the experience confident in their abilities to identify and manage racially biased patient care. Participants learned bias awareness and mitigation techniques that they planned to bring into their practice.

While a number of workshops and role-play scenarios have been published to educate about racial bias,^14–17^ to our knowledge this is the first simulation case published in *MedEdPORTAL* to address this topic. Simulation offers the realism of being in a scenario in the moment, necessitating real-time decision-making and responses. This is different from workshops, where information may be passively received or discussed, or role-play, where participants are less fully immersed.

This case is very adaptable—we were able to run it in both a simulation lab and a standard classroom. While here we have presented data from pediatric and EM providers, who are most familiar with the underlying medicine and EM environment, the case can easily be modified to make it relevant to a broader medical provider learning group. When doing so, it is important to ensure the participants are informed that the mechanism of injury of the patient's fracture pattern is well known to be benign and not concerning for nonaccidental trauma. For learners with less prior education in implicit bias or racism, facilitators may elect to begin with the introductory slide deck to introduce core concepts and then run the simulation and debriefing. Additionally, while the case was written with standardized patients in mind, due to COVID-19 restrictions, physicians with no acting background were used in the various case roles with good effect. The case required careful scripting and instructions to the standardized participants to ensure that clues of bias and authentic reactions were obtained. We found that specific instructions and role-play practice prior to the cases were very helpful. Finally, if a DEI content expert is not available for the debrief, novice facilitators may benefit from a training session (either in person or virtually) with an expert to further discuss the content and role-play possible debriefing points.

There are a few limitations of this simulation to consider. Given the nature of the case, despite careful scripting, some variations on exact wording used by standardized participants were inevitable. We do not feel that these slight variations impacted the case outcome. As with many simulations, there were a limited number of participants who were truly in the hot seat. To circumvent this limitation, facilitators could give all participants an opportunity to think about their own responses in pairs and ask them at the end of the case to immediately turn to their partner and say how they would phrase a response to a given key phrase from the biased consultant (e.g., “That's what we always do for *these* families”). Alternatively, the simulation case could be run multiple times to enable more learners to be fully immersed in the scenario. However, the case was found to be very effective by the majority of our participants whether they were physically inserted into the scenario or not. The evaluation was limited by focusing on immediate educational goals and did not include long-term data collection to investigate knowledge and self-efficacy retention. We also did not use a presurvey, which might have been useful to illustrate increases in confidence attributable to participation in the case, as a presurvey would have given away the nature of the case. Implicit bias is implicitly sneaky. Therefore, to preserve the realistic nature of the case and the participant's authentic reaction to the scenario, we elected not to use a presurvey. One could be added in future iterations.

Future directions for utilization and study of this simulation include longer-term data collection to investigate knowledge retention and inquire about bias mitigation skills employed during patient care. Additionally, the simulation could be performed with wider medical audiences to assess impact with different specialties and across medical roles. Finally, given the overwhelmingly positive reaction to the simulation session, a larger series of simulations for the DARE EM curriculum is being developed to reinforce and expand bias mitigation skills across different patient care scenarios.

## Appendices


Simulation Case.docxSimulation Images.docxSimulation HPI.docxStandardized Participant Transcripts.docxDebriefing Slides.pptxDebriefing Guide.docxPostsimulation Survey.docx
*All appendices are peer reviewed as integral parts of the Original Publication.*
