# Supplementary material for: Implicit Bias and Patient Care: Mitigating Bias, Preventing Harm
Source: MedEdPORTAL. 2023 Sep 19;19:11343. doi: 10.15766/mep_2374-8265.11343 (PMC10507144; doi:10.15766/mep_2374-8265.11343)
Supplement: Supplementary file 1 — Simulation Case.docxSimulation Images.docxSimulation HPI.docxStandardized Participant Transcripts.docxDebriefing Slides.pptxDebriefing Guide.docxPostsimulation Survey.docx [file mep_2374-8265.11343-s001.zip › B. Simulation Images.docx]

**Appendix B – Implicit Bias Simulation Images**

**Electronic Medical Record front sheet**

Child photo: Image by Terricks Noah, retrieved from [https://unsplash.com/photos/n9R0MN3XGvY on 7/5/2022](https://unsplash.com/photos/n9R0MN3XGvY%20on%207/5/2022). Creative commons license associated: <https://unsplash.com/license>

Remainder of image author owned


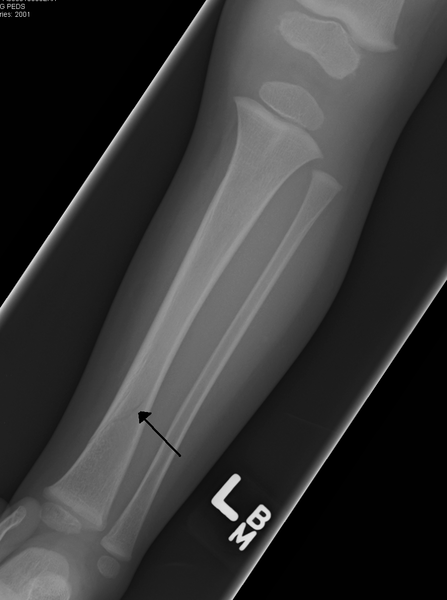


**Left tibia spiral fracture X-ray**

Image by Dr. James Heilman, retrieved from <https://commons.wikimedia.org/wiki/File:Tibfracture.png> on 7/5/2022. Creative Commons License associated:

CC BY-SA 3.0 <https://creativecommons.org/licenses/by-sa/3.0>, via Wikimedia Commons
