# Supplementary material for: Implicit Bias and Patient Care: Mitigating Bias, Preventing Harm
Source: MedEdPORTAL. 2023 Sep 19;19:11343. doi: 10.15766/mep_2374-8265.11343 (PMC10507144; doi:10.15766/mep_2374-8265.11343)
Supplement: Supplementary file 1 — Simulation Case.docxSimulation Images.docxSimulation HPI.docxStandardized Participant Transcripts.docxDebriefing Slides.pptxDebriefing Guide.docxPostsimulation Survey.docx [file mep_2374-8265.11343-s001.zip › C. Simulation HPI.docx]

**Appendix C: Implicit Bias Simulation HPI**

HPI: 18-month-old previously healthy male presenting with left leg pain. Patient going down slide on mom’s lap, mom notes his left foot got stuck on the way down, leg twisted. Patient immediately cried, subsequently would not weight bear so mom brought in. Happened about an hour prior to ED visit. Last PO 3 hours ago. No medications given.

PMH: none

PSH: none

Allergies: NKDA

Medications: none

Social History: lives with mom and older brother

Physical Exam:

Gen: alert, interactive with examiner

HEENT: atraumatic, pupils equal & reactive, oropharynx clear

Chest: regular rate and rhythm, no murmurs

Lungs: clear breath sounds bilaterally

MSK: left lower extremity without obvious deformity, tender over shin, difficult to further localize. Patient unwilling to ambulate or bear weight standing. Distal pulses 2+, sensation intact, can wiggle toes

Skin: No other injuries, no bruising

MDM: 18-month-old male presenting with inability to ambulate after injury on slide. X-ray shows left tibial spiral fracture. Discussed with orthopedics, plan for casting under sedation.
