# Supplementary material for: Implicit Bias and Patient Care: Mitigating Bias, Preventing Harm
Source: MedEdPORTAL. 2023 Sep 19;19:11343. doi: 10.15766/mep_2374-8265.11343 (PMC10507144; doi:10.15766/mep_2374-8265.11343)
Supplement: Supplementary file 1 — Simulation Case.docxSimulation Images.docxSimulation HPI.docxStandardized Participant Transcripts.docxDebriefing Slides.pptxDebriefing Guide.docxPostsimulation Survey.docx [file mep_2374-8265.11343-s001.zip › A. Simulation Case.docx]

| **Appendix A: Simulation Case**  **SIMULATION CASE TITLE: Implicit Bias & Patient Care: Mitigating Bias, Preventing Harm**  **AUTHORS: Hannah Barber Doucet, MD, MPH, Taneisha Wilson, MD, MSc, Lauren Vrablik, MD, Robyn Wing, MD, MPH**  **LEARNER AUDIENCE**: Pediatric Emergency Medicine (PEM), Emergency Medicine (EM) and pediatric residents, fellows, medical students, and faculty | |
| --- | --- |
| **PATIENT NAME: Jaylen Smith**  **PATIENT AGE: 18 months old**  **CHIEF COMPLAINT: Left leg pain**  **PHYSICAL SETTING: Pediatric Emergency Department**  **STANDARDIZED DOCTOR: Dr. Ryan Jones, 2^nd^ year orthopedic resident** | |
| **Brief narrative description of case** | An 18-month-old Black toddler presents to the ED, found to have a spiral fracture of the left tibia after getting his leg caught on the slide edge going down with mother. He is being signed out to the oncoming resident, now s/p casting under sedation. The outgoing resident instructs the oncoming resident to “just touch base with ortho about follow-up”.  The orthopedic resident will ask for DCYF to be called. |
| **Learning Objectives** | 1. Have confidence in identifying situations where implicit bias and racism may impact medical decision making 2. Express concern about racial bias using Affirm-Counter-Transform (ACT) tool 3. Express confidence in ability to prevent biased assessments from impacting patient care decisions 4. Identify appropriate and inappropriate medical scenarios in which to have concern for non-accidental trauma |
| **Critical Actions** | - Affirm that this case does not require DCYF involvement - Identify racial bias in a consultant’s assessment - Address racial bias |

| **Initial Presentation** | |
| --- | --- |
| **Overall Appearance** | Dark-skin toddler mannikin with “cast” on left lower extremity. Child in no distress. No other injuries on exam. |
| **Standardized Participants in the room at case start** | At the start: **off-going EM resident** will give sign-out, pulling up patient chart and x-ray on the computer.  Other actors:   - **Orthopedic resident**: In requesting DCYF, do not include concerns about the fall itself, the physical exam of the child, or prior injuries (of which there are none); the consultant’s reasoning is based on a “feeling” from the mom and an impression of disconnect between the parent and the consultant. - **Patient’s mother**: Mom is ready to leave the ED and is annoyed with the orthopedics resident. She feels that he was accusatory and judgmental after she was distraught that her child was hurt. She is a bit fed up with being here. - **Nurse:** May enter scenario as needed. Is a reflective listener and passes on concerns from parent. Does not offer advice about the situation. If told that the learner is calling DCYF, reacts with surprise “you’re calling DCYF for this kid?” |
| **HPI** | 18-month-old previously healthy male initially presented to the ED with leg pain after slide incident. Mom states that she was going down the slide with him and the patient caught his foot and twisted his leg on the way down. He cried immediately and was not weight bearing, so was brought in. The injury occurred about 1 hour prior to arrival. X-ray revealed a spiral fracture of the left tibia. |

| **Scenario Triggers and Progression** | | |
| --- | --- | --- |
| **Patient State** | **Trigger/Learner Action** | **Progression** |
| **State 1: Initial Presentation** | Outgoing resident is giving sign-out: sit at the computer, pull up the chart (with patient photo), and report the HPI. Show x-ray. State “They just finished his casting under sedation, I prepped the discharge, he just needs follow-up info from ortho.” | Outgoing resident answers any additional questions (if any) and leaves. |
| **State 2: Ortho resident** | Orthopedics resident will enter & say (roughly), while checking their pager “hey, we just finished on that tibia. He needs post-reduction films and can follow-up with Dr. Cruz in one week. And I’ll have you call DCYF. Thanks.” Will turn to leave.  - At the request to contact DCYF, the learner will ask for more information as to why.  (If learner doesn’t ask for more information as to why DCYF is recommended, the nurse can ask the question.) | Ortho resident response: “Well this is what we typically do for these families. And I got a weird vibe from this mom.”  If learner asks more questions, ortho resident states: “I feel like we usually recommend contacting DCYF in these cases.”  “Did you meet the family? The child is all dirty and the mom seemed like she wasn’t even paying attention to my instructions.”  The conversation may continue to go back and forth about why contacting DCYF is indicated. If pushed, the ortho resident will continue to emphasize his interactions with the family. May include:  “Mom just seemed like she wanted to get out of here. I don’t know if it’s neglect or what but come on, that just seems careless for your kid to get hurt like that. If she’s even telling the truth.”  “I don’t believe the story that mom is telling. She just doesn’t seem that reliable.”  “I don’t trust that what mom says is what really happened. She’s really defensive.”  Ortho should be checking their pager frequently, pressuring EM resident to end this discussion as they need to go deal with other consults. Can also offer that the EM resident can call them back to discuss further after speaking with the family themselves.  ***Next stage is based on learner’s response (State 3 vs. State 4)*** |
| **State 3: Refusing to call DCYF**  The learner refuses to call DCYF | Based on the discussion with orthopedics, the learner explains why they are not concerned for abuse. | If the learner brings up concerns about implicit bias, ***proceed to state 6***  If the learner does not bring up race, the ortho resident will maintain their position, re-stating discomfort with mother. The resident will extrapolate that the mother “not listening” to him makes her a bad parent, and that families “like these” need DCYF involvement. For example:  “If she can’t pay attention when I’m telling her about his cast, obviously she’s not taking care of him right. I’ve seen this too often with families like this.”  “This mom needs to know that there are consequences to not looking after her kid properly.”  “Did you see how that kid was dressed? His clothes are dirty.”  If learner remains firm, ortho resident will end with “Look I don’t have time to argue about this any more. You’re the primary team, but this is what I will be documenting as my recommendation in my note.”, then exits. CASE ENDS. |
| **State 4: Concede to calling DCYF** | Nurse will appear to say that mom is looking for an update. Nurse will inquire if the kid can go home now   - If told about DCYF, nurse reacts with surprise: “you’re calling DCYF for this kid??” | If learner continues down DCYF road, CASE ENDS |
| **State 5: Seek more details about child and family** | If the learner elects to learn more, they may:   - Read the ED note - If they want to see the patient, they will be informed that he is still rather sedate, in a cast, with no other bruises or injuries. They may examine the manikin which has no other injuries aside from casted leg. - They can talk to mom. She will respond to questions when they are specifically asked. If asked – the patient has no prior medical problems or major injuries. HPI as above. Household is mom and two kids (older child 4y/o). Grandma lives nearby and provides childcare when mom works (office manager). Additional statements may include: - “We need to be going – he has his cast now, will we be able to leave soon?” - “I already talked to the other  doctors about what happened. I’m sorry but what’s going on? We’ve had a long enough day already.” - If probing continues, mom will feel defensive, and recently was feeling judged by the orthopedic resident: “I don’t know what you’re getting at but this was an accident today and I don’t appreciate people making it out like I tried to hurt my baby. We’ve had enough and I’d really like to leave now.” | Further details should be reassuring against non-accidental trauma to the learner, and should trigger the learner to page back ortho.  If try to reach Child Protection Team – will be informed that they can try paging but someone else has been trying to get ahold of them for a while without any luck.  If try to reach pediatrician– the on-call provider calls back stating no past issues with this family. No missed appointments. Immunizations up to date. No red flags at all.  If they page back ortho, they will come back – ***go to stage 3***  If they explain to mom, they need to call DCYF – ***go to stage 4*** |
| **State 6: Ortho resident responds to racial bias concerns** | Ortho resident will respond with some surprise and back down:  “Uh, no, I don’t think it’s about race. But I mean, I guess there’s no one specific thing. Just do whatever you would normally do. Let me know when the post-reduction films are back.” | CASE ENDS |

**Ideal Scenario Flow**

The learner receives sign-out from the exiting provider. When directed by orthopedics to call DCYF for the patient, the learner immediately recognizes that this is an unusual request for this clinical scenario. The learner asks pertinent follow-up questions about why the resident feels a DCYF report is necessary, and addresses the fact that this injury and injury mechanism would not be concerning for Non-accidental Trauma (NAT). The learner would meet the patient and mother to examine the child and verify HPI with mother. The learner provides reassurance to mother as needed that they will be discharged shortly. The learner will recognize the mother’s distress after her interaction with the orthopedic resident and inquire into how she is feeling. The learner will then call back the orthopedic consultant to confirm that there is not concern for NAT and they will not report to DCYF. As the orthopedic resident escalates, the learner uses the ACT tool or other upstander tool to frame a concern about bias in this patient’s care. The orthopedic resident remains flustered and defers to the learner’s care. Case ends.

**Anticipated Management Mistakes**

1. Deference to consultant: Some learners may not immediately realize that the orthopedist’s request was not reasonable for the injury, either due to automatic deference to a consultant, or due to a deficiency in knowledge regarding toddler’s fractures. In these cases, the nurse may redirect the learner, who would at a minimum be directed to interact with the family to form their own opinion and re-discuss with orthopedics.
2. Shift decision-making responsibility to another provider (Passing the buck): Some learners may attempt to gain assistance from (appropriate) consulting services, such as social work or the hospital’s child protection team. They can be informed that these services are unavailable at the time in order to redirect the decision back to them.
3. Failure to address racial bias: Many learners may refuse to report to child welfare, but not explicitly address potential racial bias in the case. This provides a rich debriefing discussion, and learners may be given the opportunity in debriefing to reflect on how they could have approached specifically addressing bias.
