# Supplementary material for: Implicit Bias and Patient Care: Mitigating Bias, Preventing Harm
Source: MedEdPORTAL. 2023 Sep 19;19:11343. doi: 10.15766/mep_2374-8265.11343 (PMC10507144; doi:10.15766/mep_2374-8265.11343)
Supplement: Supplementary file 1 — Simulation Case.docxSimulation Images.docxSimulation HPI.docxStandardized Participant Transcripts.docxDebriefing Slides.pptxDebriefing Guide.docxPostsimulation Survey.docx [file mep_2374-8265.11343-s001.zip › E. Debriefing Slides.pptx]

## Slide 1
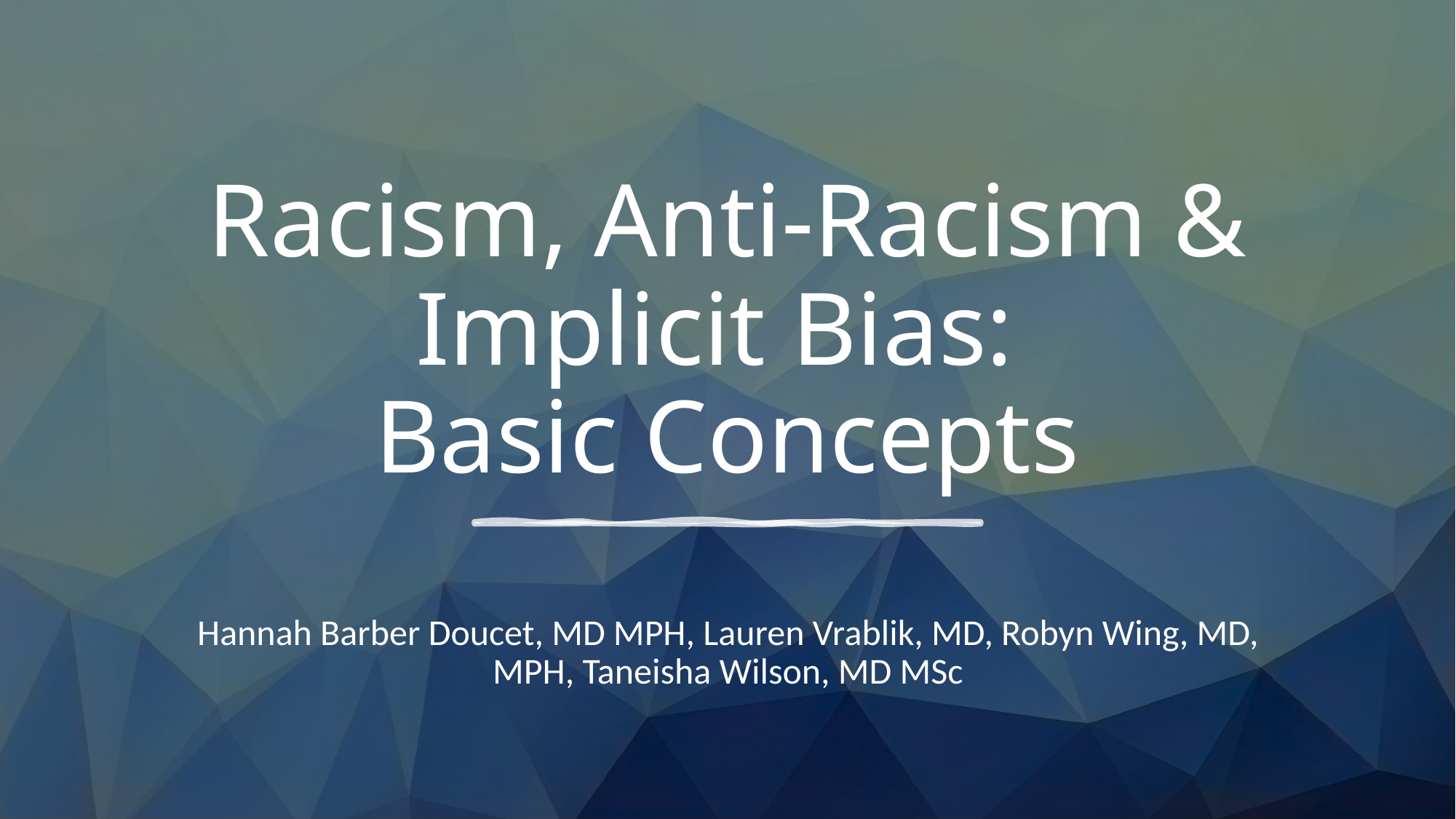

# Racism, Anti-Racism & Implicit Bias: Basic Concepts
Hannah Barber Doucet, MD MPH, Lauren Vrablik, MD, Robyn Wing, MD, MPH, Taneisha Wilson, MD MSc

## Slide 2
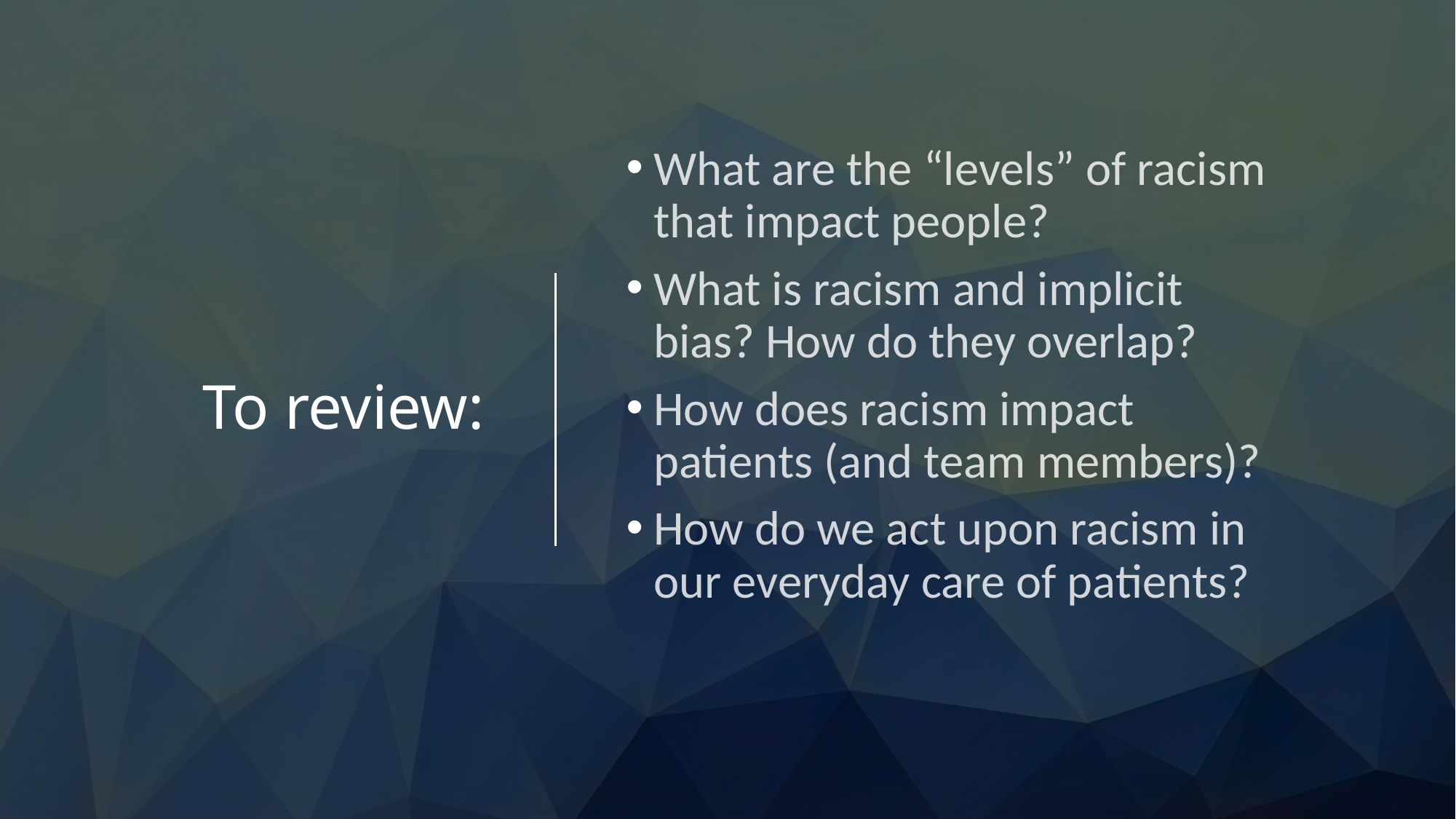

# To review:
What are the “levels” of racism that impact people?
What is racism and implicit bias? How do they overlap?
How does racism impact patients (and team members)?
How do we act upon racism in our everyday care of patients?

## Slide 3
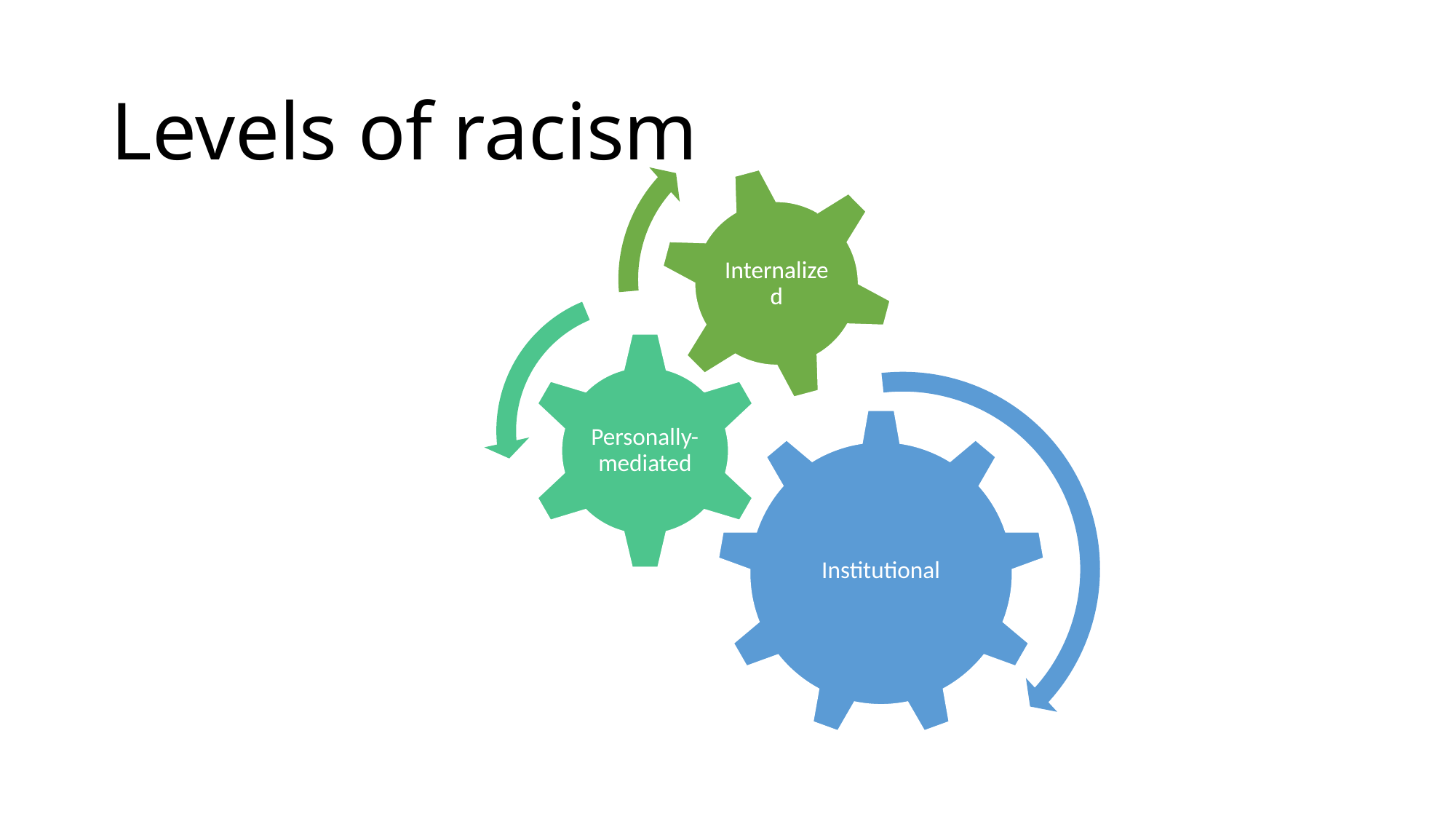

# Levels of racism

## Slide 4
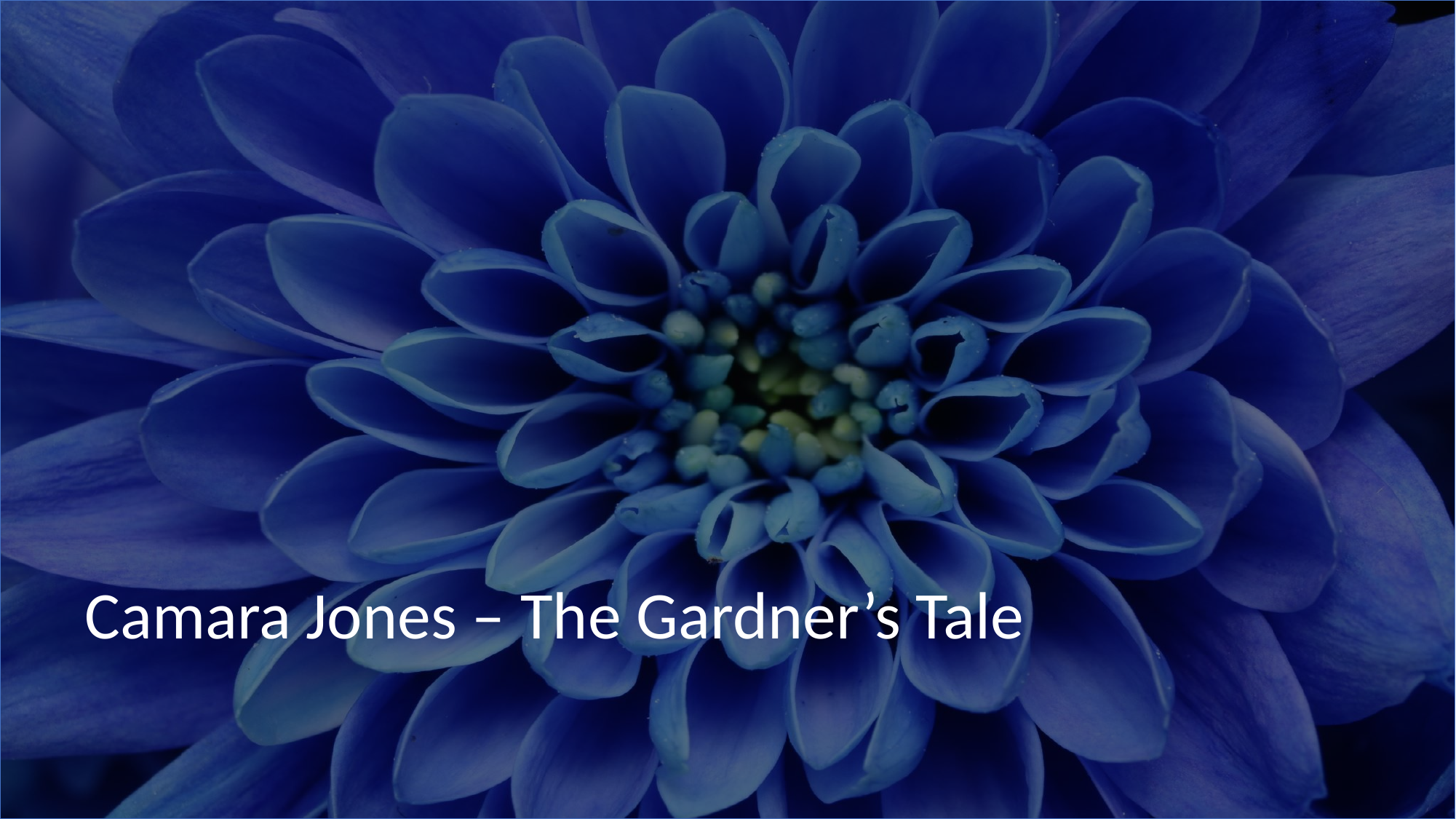

Camara Jones – The Gardner’s Tale

## Slide 5
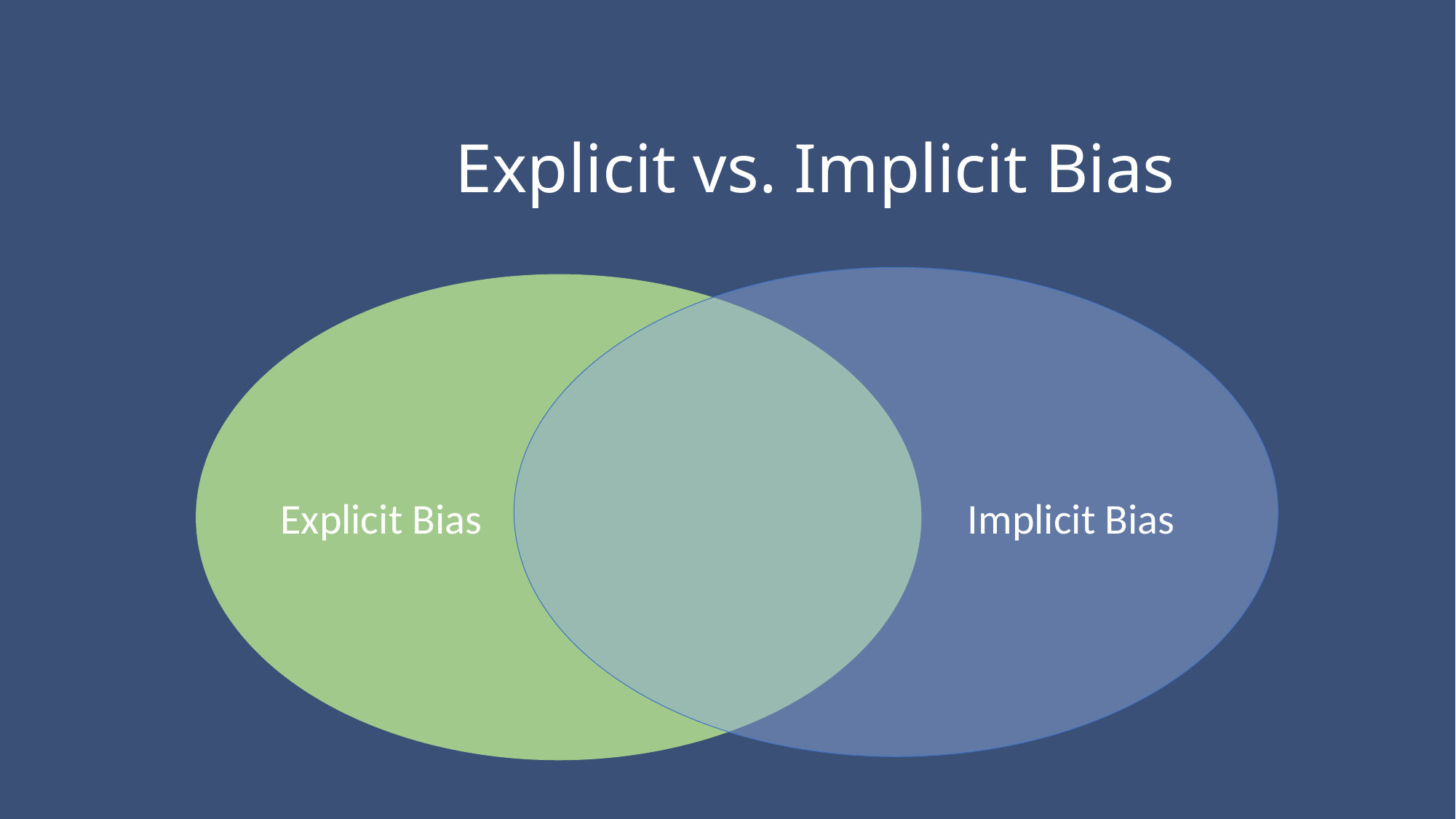

# Explicit vs. Implicit Bias
Explicit Bias
Implicit Bias

## Slide 6
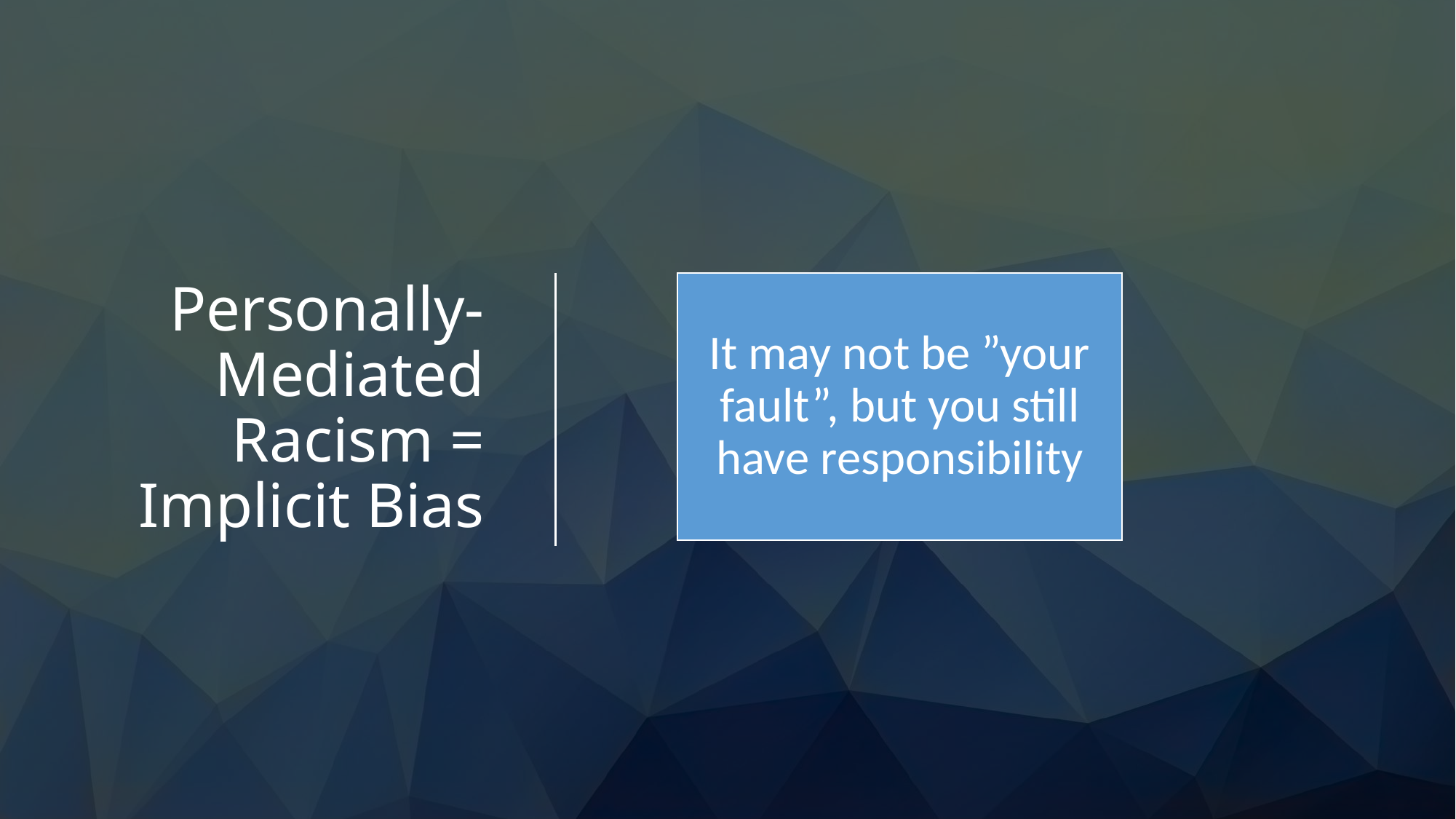

# Personally-Mediated Racism = Implicit Bias

## Slide 7
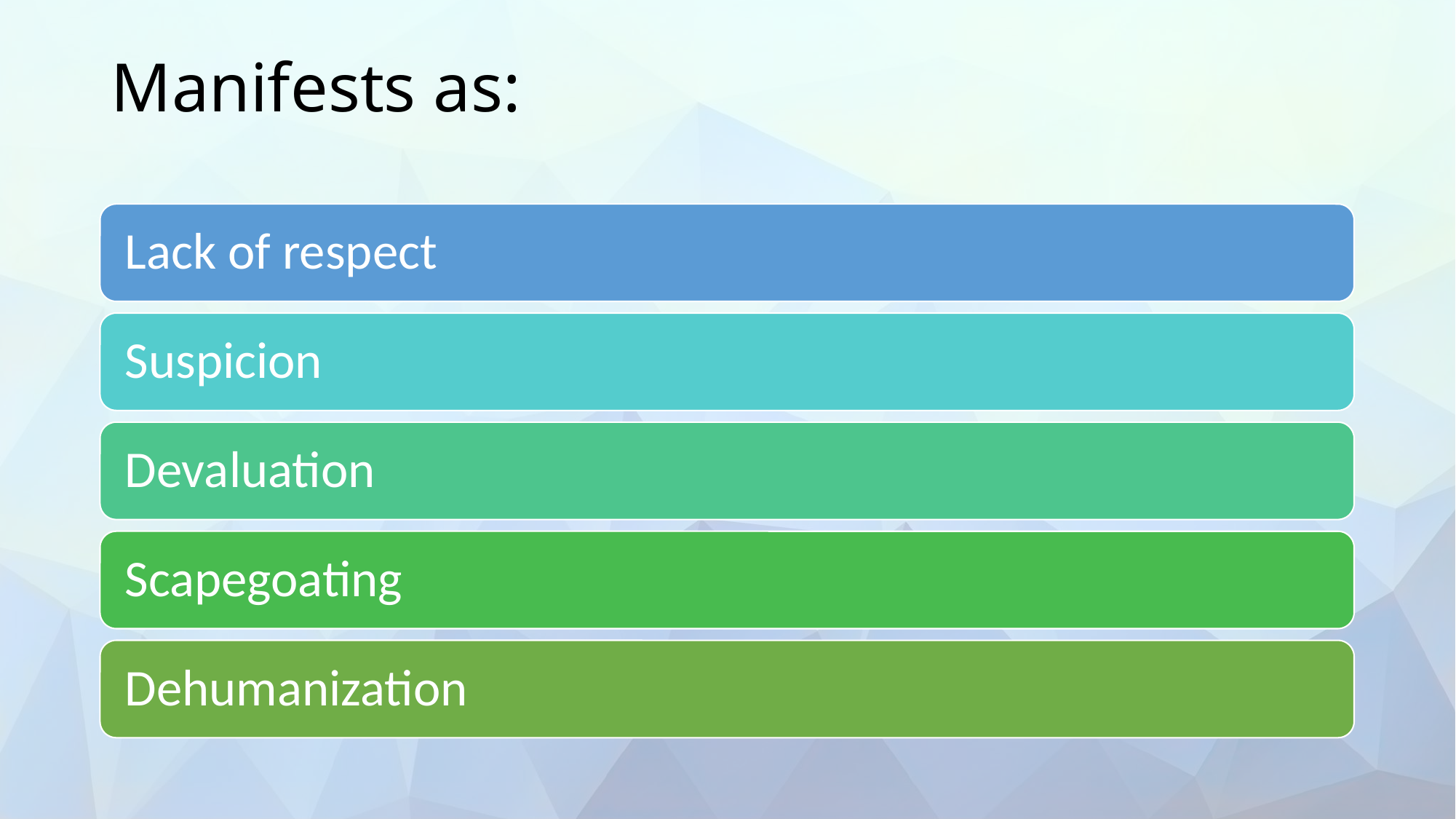

# Manifests as:

## Slide 8
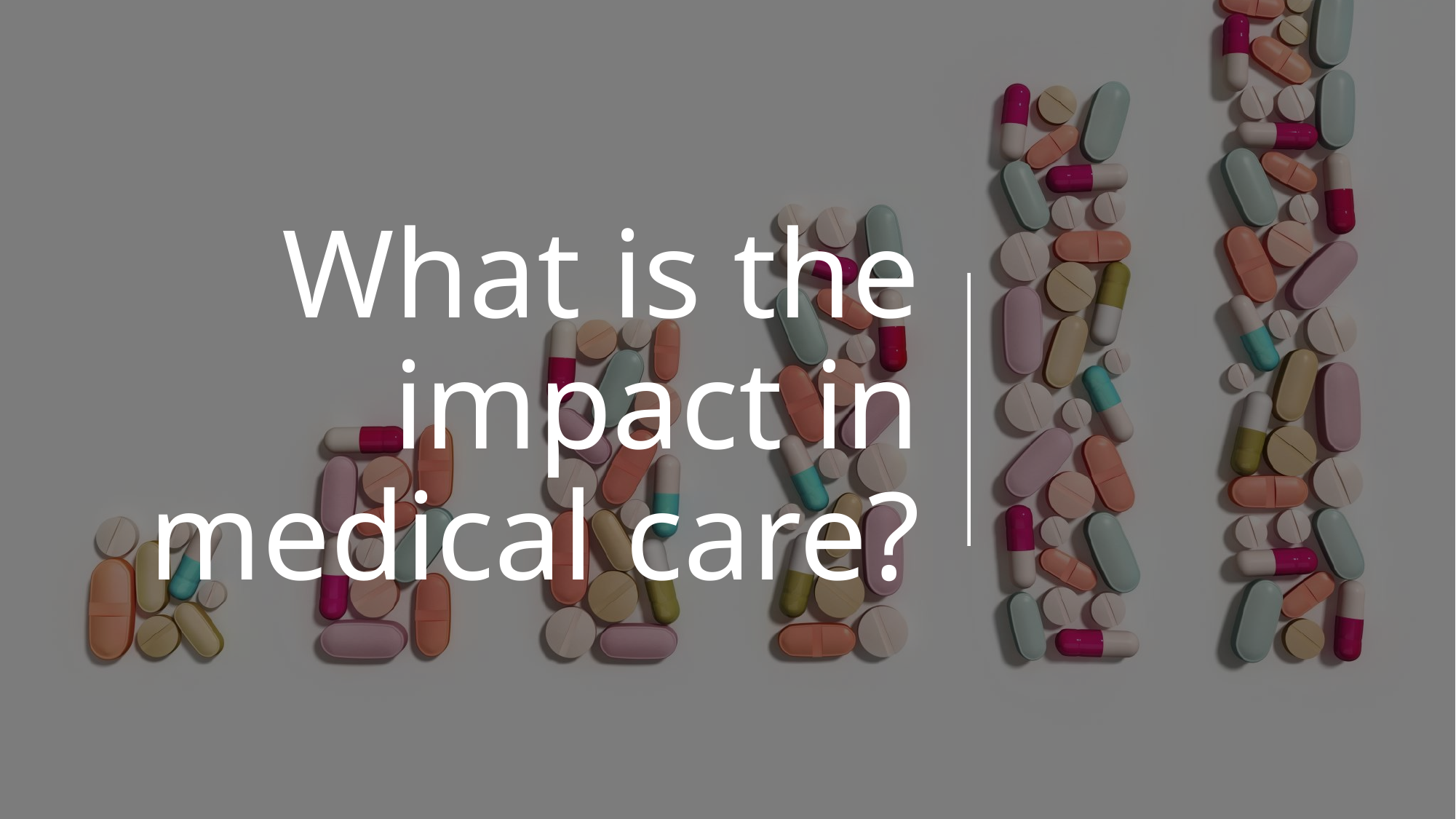

# What is the impact in medical care?

## Slide 9
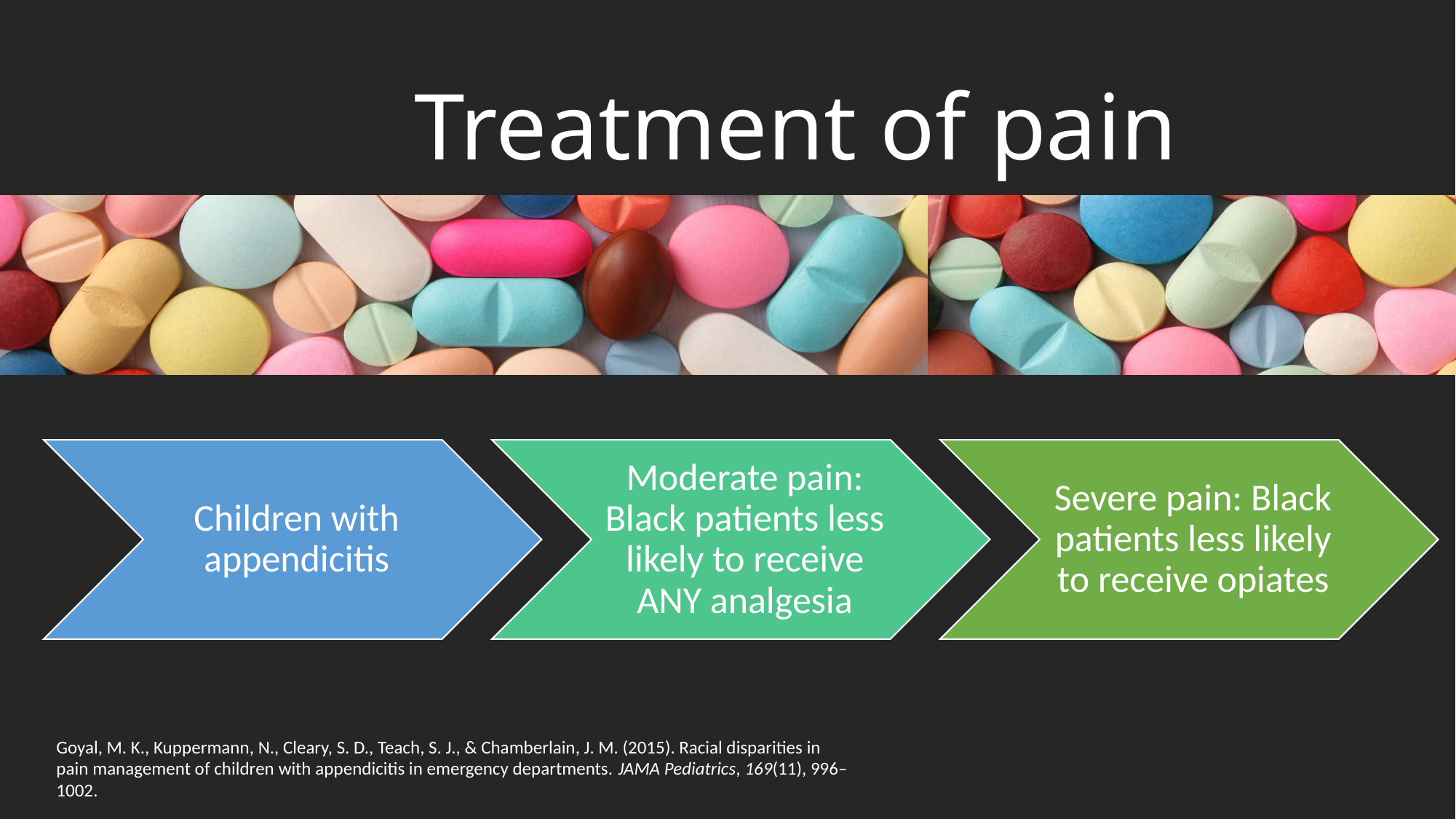

# Treatment of pain
Goyal, M. K., Kuppermann, N., Cleary, S. D., Teach, S. J., & Chamberlain, J. M. (2015). Racial disparities in pain management of children with appendicitis in emergency departments. JAMA Pediatrics, 169(11), 996–1002.

## Slide 10
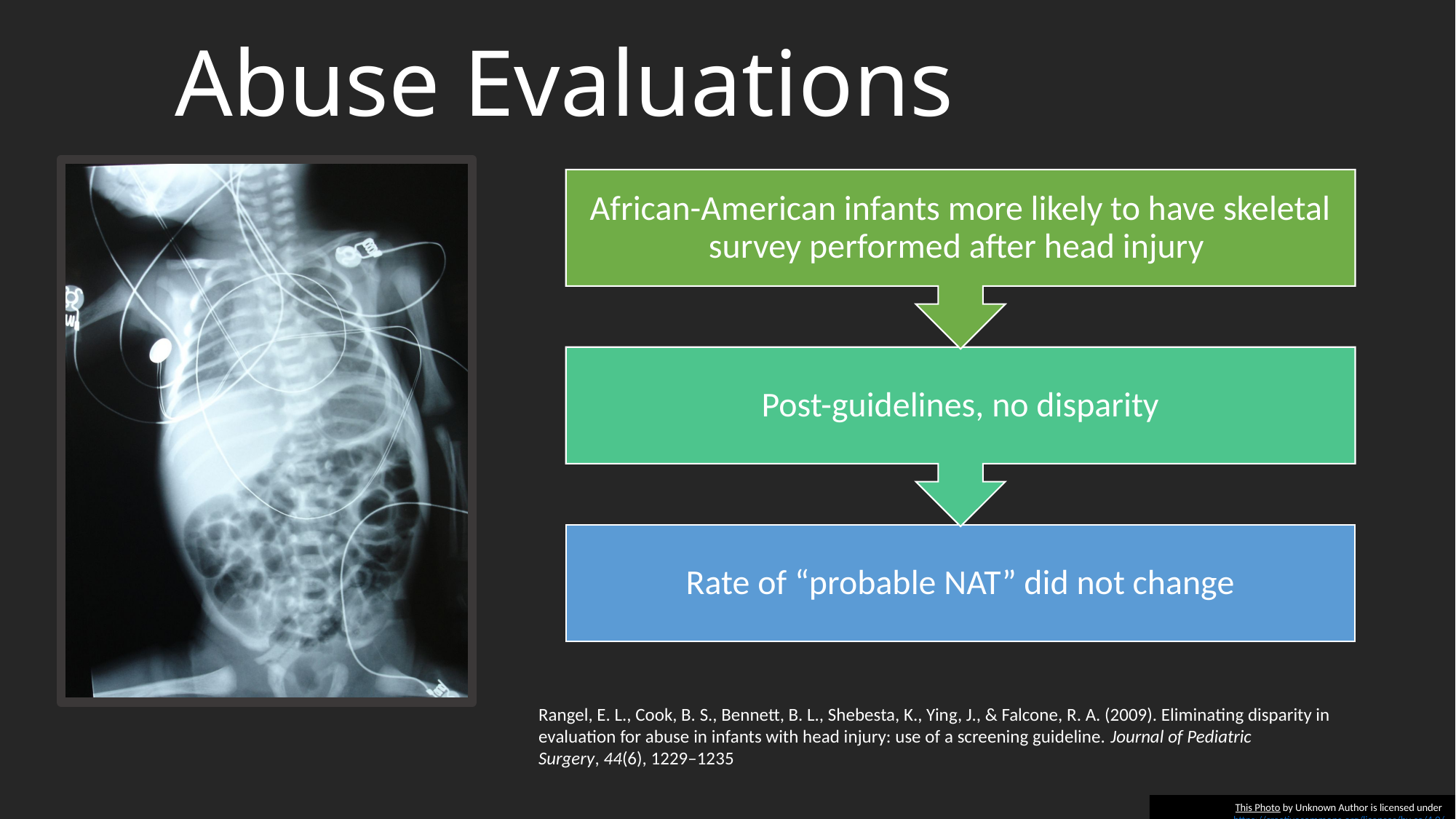

# Abuse Evaluations
Rangel, E. L., Cook, B. S., Bennett, B. L., Shebesta, K., Ying, J., & Falcone, R. A. (2009). Eliminating disparity in evaluation for abuse in infants with head injury: use of a screening guideline. Journal of Pediatric Surgery, 44(6), 1229–1235
This Photo by Unknown Author is licensed under https://creativecommons.org/licenses/by-sa/4.0/

## Slide 11
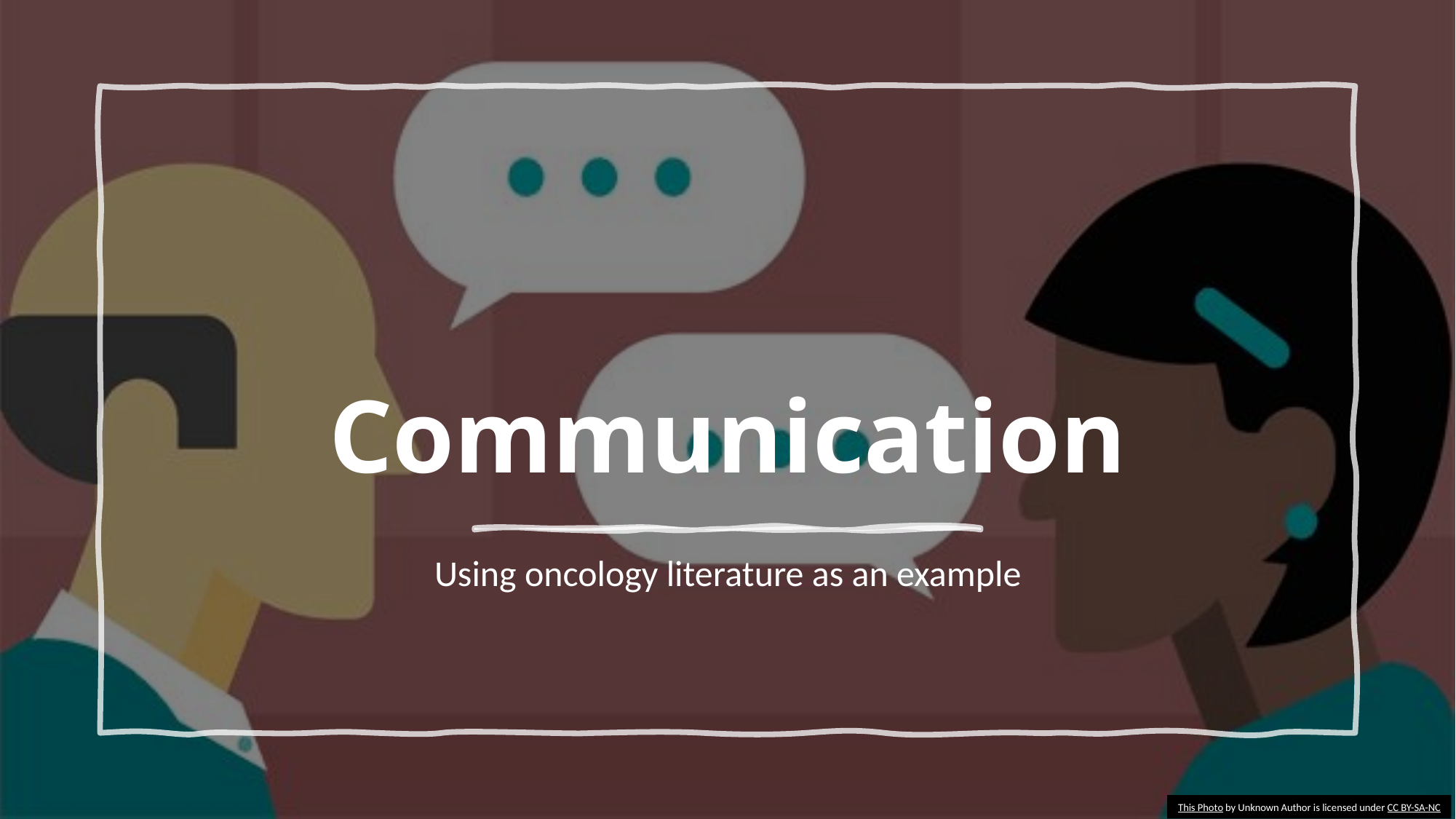

# Communication
Using oncology literature as an example
This Photo by Unknown Author is licensed under CC BY-SA-NC

## Slide 12
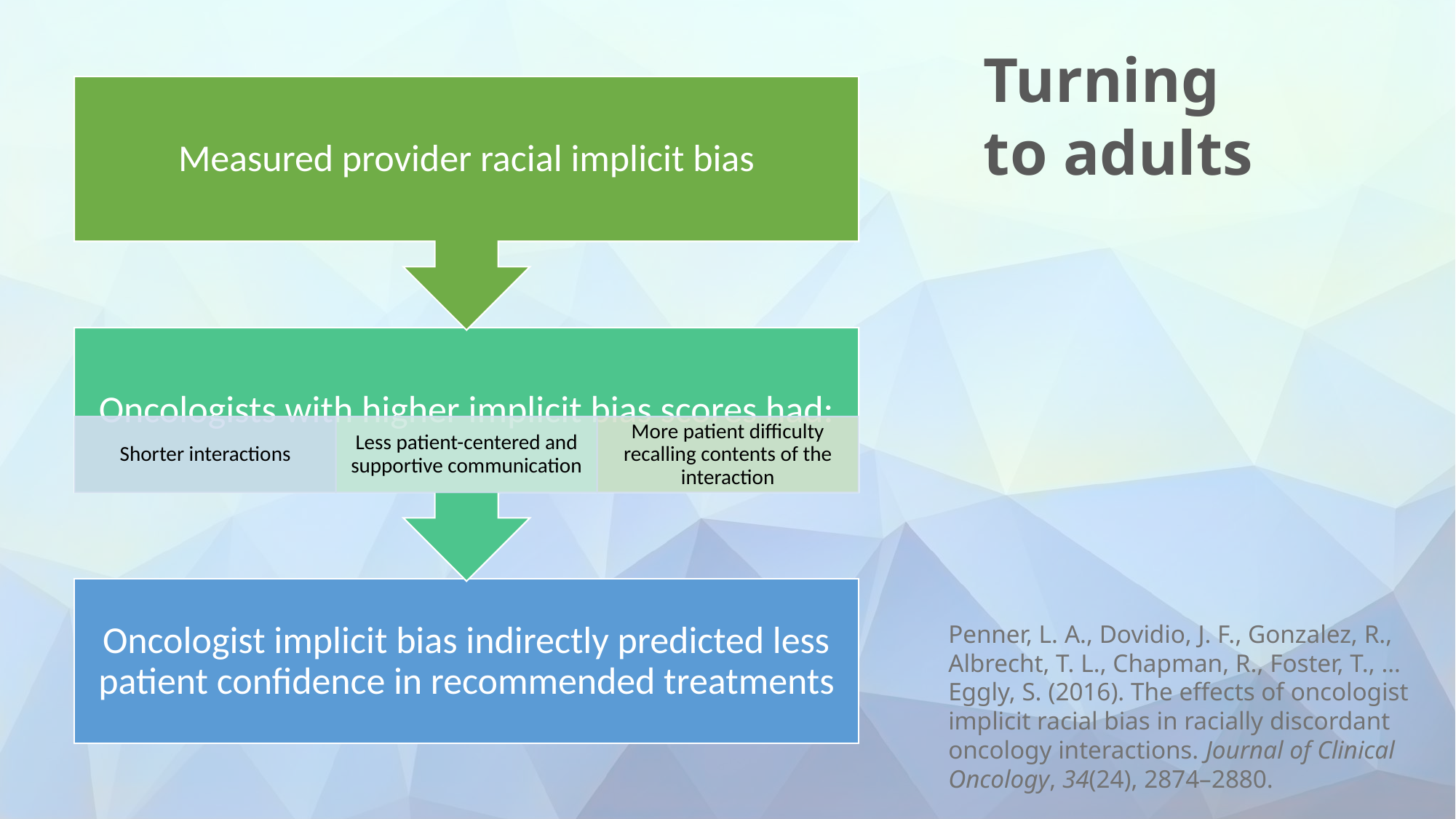

Turning to adults
Penner, L. A., Dovidio, J. F., Gonzalez, R., Albrecht, T. L., Chapman, R., Foster, T., … Eggly, S. (2016). The effects of oncologist implicit racial bias in racially discordant oncology interactions. Journal of Clinical Oncology, 34(24), 2874–2880.

## Slide 13
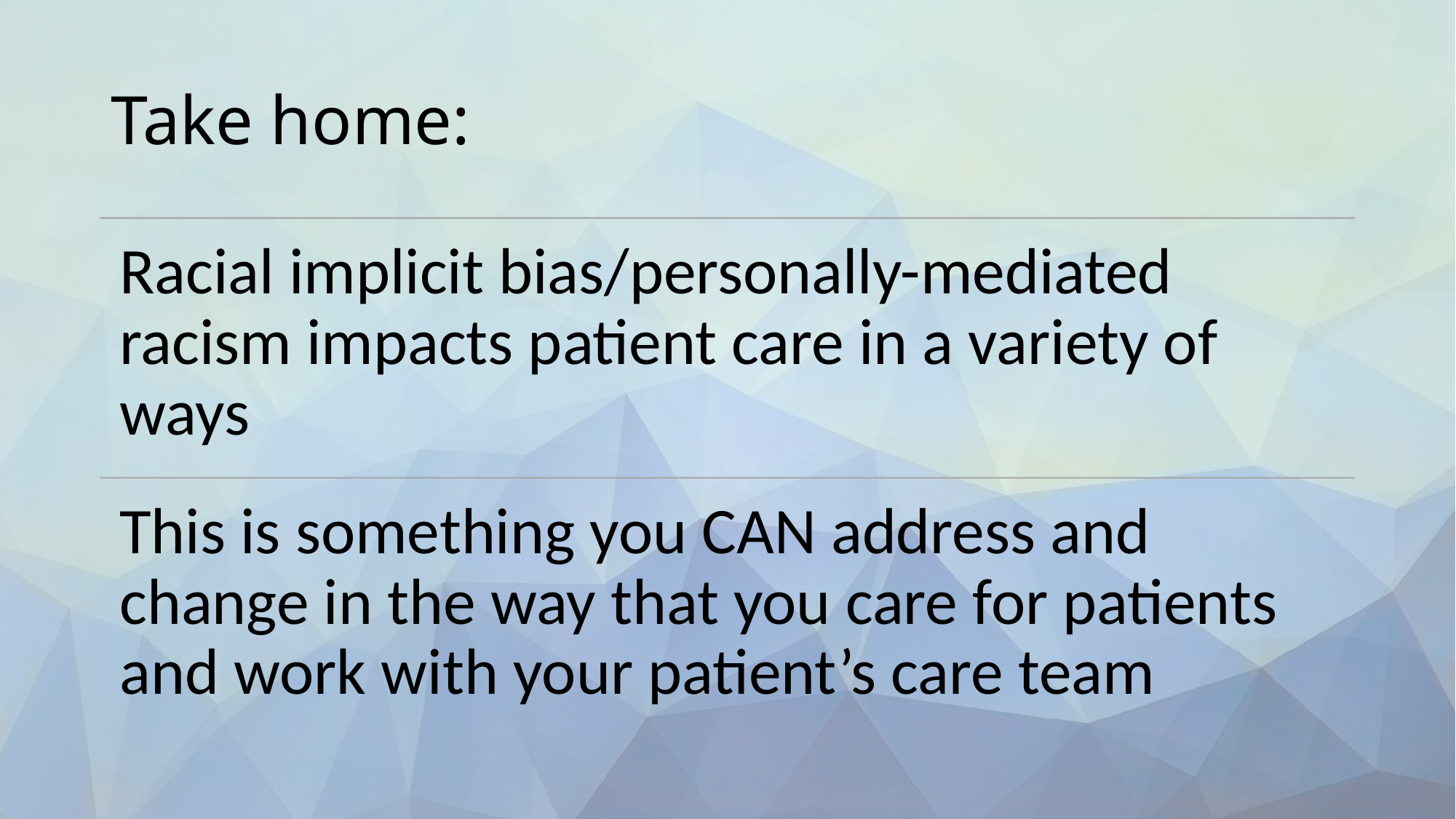

# Take home:
