# Supplementary material for: Implicit Bias and Patient Care: Mitigating Bias, Preventing Harm
Source: MedEdPORTAL. 2023 Sep 19;19:11343. doi: 10.15766/mep_2374-8265.11343 (PMC10507144; doi:10.15766/mep_2374-8265.11343)
Supplement: Supplementary file 1 — Simulation Case.docxSimulation Images.docxSimulation HPI.docxStandardized Participant Transcripts.docxDebriefing Slides.pptxDebriefing Guide.docxPostsimulation Survey.docx [file mep_2374-8265.11343-s001.zip › F. Debriefing Guide.docx]

**Appendix F. Implicit Bias Debriefing Guide**

1. **Introduction:**

*We are now going to take the next 15 minutes to debrief. This part of our session is actually more important than the actual case. As a reminder, our ground rules are as follows:*

- *This is meant to be a safe learning environment*
- *Simulation is a formative, interdisciplinary learning environment - no one is being evaluated (except the simulation instructors because we constantly evaluate ourselves on how to provide better educational sessions).*
- *We all agree to the basic assumption that everyone here is intelligent, well trained, and cares about doing their best.*
- *Thus, we all agree to the highest standard of professional conduct and courtesy to our colleagues in this debrief and in any further discussions about this session.*

**2. Emotional Experience:**

*That was a challenging/stressful/difficult case. Cases that bring up bias can be really tricky to handle. Let’s talk about how things went.*

*How did that feel to do? How did it go?*

**3. Case Summary:**

*Can someone describe what was going on in this case?*

Discussion of implicit bias/racism vs explicit bias/racism:^[[1]](#footnote-1)^

- Explicit biases are set of conscious beliefs that you would be able to report about yourself.
- Implicit biases are unconscious, automatic and are not recognized or acknowledged by the individual.
- Can imagine other similar cases where implicit/explicit biases of providers have an impact on patient care. While this was not the exact case here, there are likely many potential cases of microaggressions learners may have witnessed among health care professionals.

(Consider using slides in Appendix E to ground discussion of implicit bias/racism)

**4. Medical Knowledge (as needed for learners):**

Review toddler’s fractures:^[[2]](#footnote-2)^

- Toddler’s fractures are non-displaced spiral fractures of the tibia, with an intact periosteum. They are specific to young mobile children, generally ages 9 months to 3 years. Common causes are a twisting motion, which can be minor – such as getting a foot caught while going down a slide, or stepping and twisting/falling. They may also present with leg pain/difficulty ambulating without a known mechanism, since in this age group falling is common and developmentally appropriate, and the exact moment of injury may be difficult to pinpoint. The fracture may be difficult to appreciate on x-ray. Treatment includes casting and follow-up.

Review when to be concerned for non-accidental trauma (NAT), such as the TEN-4 mnemonic^[[3]](#footnote-3)^ and other basic teachings:^[[4]](#footnote-4)^

- Findings concerning for NAT include bruising in particular regions or patterns, burns in particular patterns, repeated suspicious injuries, or any injury that does not match the history given and/or the developmental stage of the child. Identifying injuries that trigger concern for NAT is especially important in young children who may not be able to report the history of injury themselves, and are also at higher risk for NAT.
- TEN-4 outlines those bruising areas & ages most frequently associated with NAT – for those under 4 years of age, the trunk, ears or neck; and any bruising under 4 months of age. Other areas of concern include any intraoral injury, GU bruising/injury, and patterned bruising. Burns that are concerning for NAT include a pattern of immersion burn - a clean demarcation line of burn edge around an extremity or the buttocks, as opposed to a splatter pattern which is commonly seen with accidental burns.
- It may be useful at this point to emphasize that when participants *do* have concerns for NAT, they are mandated reporters and should *absolutely* be pursuing these concerns. If they are unsure, there are often other resources at their disposal, such as conferring with the patient’s pediatrician, or discussing with their local child abuse specialist (if applicable).
- Keep in mind that while your impression of the family is important, this is the part of the evaluation that is most open to bias. Families may be nervous or upset for a variety of reasons in the emergency department. Your impression of the family is a good opportunity for self-evaluation of if bias may be impacting your assessment (due to race or a variety of other factors).

**5. Bias Mitigation Techniques:**

*What approaches did [the participant] utilize to handle this case?*

*How might you handle this scenario?*

Keep in mind that there are a number of appropriate approaches. This should include addressing clearly to the consultant your assessment that this injury is clearly consistent with toddler’s fractures and you/your team do not have concerns for NAT. This should also include questions to the consultant about why they are concerned. When a potentially biased reasoning is presented, it is useful to highlight potential bias, particularly in a scenario where there is a known disparity. For best collaboration, it is useful to do this in a clear, non-accusatory manner. There are number of upstander tools that work well, and participants will find with time what works best for their personal communication styles.^[[5]](#footnote-5)^

An upstander tool that works well in this type of scenario, when addressing a clear impact on patient care, is to try something slightly more involved and structured, like ACT:

**ACT Acronym for addressing bias**^[[6]](#footnote-6)^

**A: affirm** shared values and presumed good intentions on the part of all parties;

**C: counter** and explain why there is a problem in this specific situation and give historical context. State bias explicitly and non-judgmentally, potentially the hardest part;

**T: transform** and propose a solution.

For example: “I know we all want to ensure every child is safe. However, based on the history and injury of this child, I’m not concerned for abuse. Black families tend to get over-reported to child protection, and I worry about racial bias influencing our decision making. I’m happy to meet the family and be an additional set of eyes on the situation. Were there any specific concerns you had?”

**6. Further Reflection (if time):**

*What microaggressions or implicit bias related actions and statements have you witnessed in your practice? Have you ever responded, and if so, how?*

*This case was built around an existing disparity in pediatrics, in rates of child welfare evaluation, referral and involvement. What racial disparities exist in your field? How might they come up in individual patient encounters? What tools can you envision using to either monitor your own practice or help other providers you work with?* [in larger groups, can frame these questions as a Think, Pair, Share exercise]

1. <https://www.nationalequityproject.org/frameworks/implicit-bias-structural-racialization>

   https://nmaahc.si.edu/learn/talking-about-race/topics/being-antiracist [↑](#footnote-ref-1)
2. https://dontforgetthebubbles.com/toddler-fracture/ [↑](#footnote-ref-2)
3. Pierce MC, Kaczor K, Aldridge S, O’Flynn J, Lorenz DJ. Bruising Characteristics Discriminating Physical Child Abuse From Accidental Trauma. *Pediatrics*. 2010;125(1):67-74. doi:10.1542/PEDS.2008-3632 [↑](#footnote-ref-3)
4. https://www.acepnow.com/article/ten-4-faces-p-a-mnemonic-to-help-you-spot-signs-of-child-abuse/ [↑](#footnote-ref-4)
5. Facilitators should be versed in microaggressions and intervention techniques. If they need review prior to the session, there are many sessions on <https://www.mededportal.org/anti-racism> with facilitator guides that review this material. Two examples with basic microaggression examples and responses include “Interrupting Microaggressions in Health Care Settings: A Guide for Teaching Medical Students” and “Taking the VITALS to Interrupt Microaggressions” [↑](#footnote-ref-5)
6. As initially described by the Center for Social Inclusion in New York City [↑](#footnote-ref-6)
