# Supplementary material for: Implicit Bias and Patient Care: Mitigating Bias, Preventing Harm
Source: MedEdPORTAL. 2023 Sep 19;19:11343. doi: 10.15766/mep_2374-8265.11343 (PMC10507144; doi:10.15766/mep_2374-8265.11343)
Supplement: Supplementary file 1 — Simulation Case.docxSimulation Images.docxSimulation HPI.docxStandardized Participant Transcripts.docxDebriefing Slides.pptxDebriefing Guide.docxPostsimulation Survey.docx [file mep_2374-8265.11343-s001.zip › G. Postsimulation Survey.docx]

**Appendix G: Implicit Bias Post-Simulation Survey**

What is your current level of training?

☐ PGY 1 ☐ PGY 2 ☐ PGY 3 ☐ PGY 4

☐ PGY 5 ☐ PGY 6 ☐ PGY 7 ☐ Attending Physician

☐ Other _______________________

Do you work primarily in:

☐ Adult Emergency Medicine ☐Pediatric Emergency Medicine ☐Both

**Please rate your agreement with the following statements:**

|  | Strongly Disagree | Disagree | Neither Agree nor Disagree | Agree | Strongly Agree |
| --- | --- | --- | --- | --- | --- |
| This case is relevant to my work. | □ | □ | □ | □ | □ |
| This case was realistic. | □ | □ | □ | □ | □ |
| This case was effective in teaching bias communication skills. | □ | □ | □ | □ | □ |
| The debrief created a safe environment. | □ | □ | □ | □ | □ |
| The debrief promoted reflection and team discussion. | □ | □ | □ | □ | □ |

**After participating in this session, how confident are you in your ability to:**

|  | Very Not confident | Not confident | Neutral | Confident | Very Confident |
| --- | --- | --- | --- | --- | --- |
| Identify situations where implicit bias and racism may impact medical decision making | □ | □ | □ | □ | □ |
| Express concern about racial bias using Affirm-Counter-Transform (ACT) tool | □ | □ | □ | □ | □ |
| Prevent biased assessments from impacting patient care decisions | □ | □ | □ | □ | □ |

What did you take away from this case and/or how will it change your practice?

____________________________________________________________________

____________________________________________________________________

____________________________________________________________________

What specific changes would you make to improve this scenario?

____________________________________________________________________

____________________________________________________________________

Other comments or suggestions:

____________________________________________________________________

____________________________________________________________________

____________________________________________________________________

**Thank you for taking the time to complete this survey!**
