# Supplementary material for: Implicit Bias and Patient Care: Mitigating Bias, Preventing Harm
Source: MedEdPORTAL. 2023 Sep 19;19:11343. doi: 10.15766/mep_2374-8265.11343 (PMC10507144; doi:10.15766/mep_2374-8265.11343)
Supplement: Supplementary file 1 — Simulation Case.docxSimulation Images.docxSimulation HPI.docxStandardized Participant Transcripts.docxDebriefing Slides.pptxDebriefing Guide.docxPostsimulation Survey.docx [file mep_2374-8265.11343-s001.zip › D. Standardized Participant Transcripts.docx]

**Appendix D. Standardized Participant Transcripts**

**Orthopedics Resident: Standardized Participant Role**

You are the orthopedics resident on call, and have been consulted in the pediatric emergency department for a patient with a spiral fracture of the tibia. You have reduced the fracture and casted the extremity, and attempted to discuss precautions and follow up instructions with the patient’s mother. However, she seems distracted and more focused on getting discharged, which you find suspicious given the injury pattern. Your conversation with her did not go well – she becomes increasingly short and frustrated. You also notice the child’s clothing is dirty. Given the combination of this parental behavior and appearance of the child in the setting of an injury, you decide that this case needs to be referred to Child Protective Services (CPS). There is racist implicit bias in your judgement call, but this is subconscious.

While this has all been taking place, you have missed multiple pages from other departments that urgently need your attention prior to you needing to give sign out to the oncoming resident within the next few minutes. You go to find the emergency department resident to quickly wrap up the plan of care.

**Notes for the SP:**

- When discussing the care plan with the learner you are feeling stressed from your previous encounter and also stressed about the amount of work you need to get done in a short time before your relief comes on shift. You speak rapidly and are checking your pager as you talk, distracted.
- When you initially approach the learner, refer to the patient you were taking care of as “The tibia fracture.” Tell them that you completed the reduction and casting, that the patient should follow up in the clinic next week. Then say as you begin walking away, “Oh and call this case in to Child Protective Services, thanks!”
  - If the learner tells you to wait, try not to engage in conversation. Tell them you’re busy and need to return other cages and can’t discuss the case right now.
  - If the learner backs off at this point, walk away. If they continue to persist say “Have you even seen this kid yet? Why don’t you go talk to the family and you’ll see what I mean.”
- After the learner talks to the family, they may ask for you (the ortho resident) to return. If so, come back into the sim and re-engage in discussion about Child Protection. If they do not specifically ask, you can stand nearby and say, “I’m wrapping up my shift, do we need to finalize anything about the plan for this case?”
- If they concede to calling CPS say, “Great thanks I’ll let the oncoming Ortho team know the plan.”
- If they continue to probe into your decision making without explicitly bringing up implicit bias around race, give several excuses, emphasizing intangible details of your interpersonal interactions:
  - “Well, this is what we typically do for these families. And I got a weird vibe from this mom.”
  - “I feel like we usually recommend contacting DCYF for these families.”
  - “Mom just seemed like she wanted to get out of here. I don’t know if it’s neglect or what but come on, that just seems careless for your kid to get hurt like that. If she’s even telling the truth.”
  - “Did you meet the family? The child is all dirty and the mom seemed like she wasn’t even paying attention to my instructions.”
  - “If she can’t pay attention when I’m telling her about his cast, obviously she’s not taking care of him right. I’ve seen this too often with families like this.”
  - “This mom needs to know that there are consequences to not looking after her kid properly.”
  - “I don’t believe the story that mom is telling. She just doesn’t seem that reliable.”
  - “I don’t trust that what mom says is what really happened. She’s really defensive.”
- Ortho should be checking their pager frequently, pressuring the learner to end this discussion as they need to go deal with other consults.
- If learner remains firm against contacting CPS, ortho resident will end with “Look I don’t have time to argue about this anymore. You’re the primary team, but this is what I will be documenting as my recommendation in my note.”
- If the learner explicitly brings up racial bias Ortho resident will respond with some surprise and back down saying, “Uh, no, I don’t think it’s about race. Just do whatever you would normally do. Let me know when the post-reduction films are back.”

**Mother: Standardized Participant Role**

You are the mother of an 18-month-old boy, Jayden. You brought him to the emergency department (ED) today after he caught his foot and twisted his left leg while going down a slide at the playground. He was crying immediately and was not bearing weight on his leg. He had an x-ray that revealed a spiral fracture of his left tibia.

The initial provider (other SP who is the orthopedics resident) finished casting Jayden’s leg and you are waiting for the all-clear after his sedation for the casting. The nurse just let you know that since he is now sitting up, she’s going to grab him a popsicle and the doctor to get the discharge read.

You don’t tell the nurse, but you’re annoyed with the initial provider (orthopedics) because you feel that they were accusatory and judgmental after you were distraught that your child was hurt.

**Notes for the SP:**

- You will respond easily and clearly to questions when they are specifically asked. Take at least one opportunity to mention that you were under the impression you would be leaving soon. You are worried about the time because you need to get Jayla from pre-K, but this visit is running longer than they initially said. This may include:
  - If the learner does not immediately engage you may ask, "We need to be going - he has his cast now, will we be able to leave soon?"
  - If they ask for additional information you say "I already talked to the other doctors about what happened. I'm sorry but what's going on? We've had a long day and I really do have run."
- If asked about Jayden’s past medical history and social history, this includes: Jayden has no prior medical problems or major injuries. Your family includes Jayden’s 5-year-old sister, Jayla, and your husband Joe who is an accountant. Your mother and siblings live nearby and provides childcare when you work as an office manager in a cardiologist’s office. You live in the same zip code as the health center. You have robust family support.
- If asked about your understanding of Jayden’s injury/care from the orthopedic resident, you are able to report back his fracture, that you should keep the cast dry, and follow up with orthopedics in one week (full recall of teaching).
- If the learner is pressing for more than a few questions or gives an indication that there is a hold-up in discharge, you may ask how long things will take (if not brought up prior) and request that they call Jayden's pediatrician for additional information if there is any medical concern or concern about him since you have been with the same pediatrician for years with Jayla and now Jayden.
  - If they tell you it’s going to be longer because they must call Child Protective Services, you call your mother to pick Jayla up from school.
- If the learner addresses your distress directly (your statements of needing to leave, how you are answering all questions calmly and directly but clearly frustrated with the process), your response should depend on how this information is delivered. *This discussion may or may not occur depending on the learner*.
  - If it does not feel like an empathetic delivery in either body language (learner standing across the room, not making eye contact, etc.) or tone of delivery (dismissive/accusatory), brush off this inquiry quickly – for example, “I just need to pick up my daughter at school.”
  - If delivered empathetically that makes this learner feel trustworthy, may disclose that you felt accused and talked down to by the orthopedic resident. This will still be a hesitatingly delivered complaint and will not lead further – can close with “I don’t really want to get into it.”
- If the learner brings up concerns for non-accidental trauma/child abuse, or a need to call Child Protective Services, your response should depend on how this information is delivered. *This discussion may or may not occur depending on the learner*.
  - If you feel the learner is confrontational or judgmental when they mention child abuse or Child Protective Services, become defensive and less interested in discussing anything with them. You may also say, "I don't know what you're getting at, but this was an accident today and I don't appreciate people making it out like I tried to hurt my baby. We've had enough and I'd really like to leave now."
  - If the learner is compassionate and straightforward, remain engaged and interested. You may express some mild distress that this is a concern.
  - If the provider tells you that they have to report your case to Child Protective Services, be sure to ask the reason.
  - If the learner pre-emptively includes something to the effect of “this is a requirement based on the injury” you add “the nurse told me that this happens to kids all the time, and they get these cases from the playground almost every day. Do you call Child Protective Services on all of them?”; you may also add “is this because of the color of our skin?” *or* “I looked it up on Wikipedia and it says it’s a really common injury and the example they use is exactly what happened to Jayden today.”
- If the learner declines to file a Child Protective Services report and shares that with you, but they do not address racial bias in the case, you may still express concern. You may even ask “Why was Child Protective Services even considered? The nurse told me that there’s at least one or two children every day with the same injury. Do you report on all of them?”
